# Diagnostic anténatal d’un hémangiome du membre supérieur

**DOI:** 10.11604/pamj.2016.25.192.10909

**Published:** 2016-11-25

**Authors:** Amira Ayachi, Mechaal Mourali

**Affiliations:** 1 Service de Gynécologie et Obstétrique, Unité De Diagnostic Anténatal, Faculté de Médecine de Tunis, Université Tunis El Manar, CHU Bougatfa, Bizerte, Tunisie

**Keywords:** Diagnostic anténatal, hémangiome, anémie foetale, Prenatal diagnosis, hemangioma, fetal anemia

## Image en médecine

Nous rapportons le cas d’une patiente G3P3, adressée à notre Unité de diagnostic anténatal pour anasarque fœtoplacentaire à un terme de 31 SA associée à une rupture prématurée des membranes. L’échographie réalisée montrait un fœtus en anamnios, une anasarque fœtale, une mesure du pic systolique de vélocité au niveau de l’artère cérébrale moyenne >1.5 MoM et un épaississement cutané du membre supérieur droit évoquant une tumeur fœtale vascularisée. Le doppler énergie montrait le départ de vaisseaux de l’aorte communiquant avec l’artère sous clavière droite (A,B). Deux heures après l’hospitalisation, la survenue d’une souffrance fœtale aigue a imposé un accouchement par césarienne. L’examen du nouveau-né de sexe masculin montrait une tumeur vascularisée prenant et déformant tout le membre supérieur droit, allant jusqu’à la partie haute du thorax (C,D). Un thrill a été objectivé à la palpation, confirmé par la présence d’un souffle au niveau de la tumeur, témoin de shunts artério-veineux. Le décès du nouveau-né est survenu 30 minutes après la naissance malgré une réanimation néonatale active.

**Figure 1:**
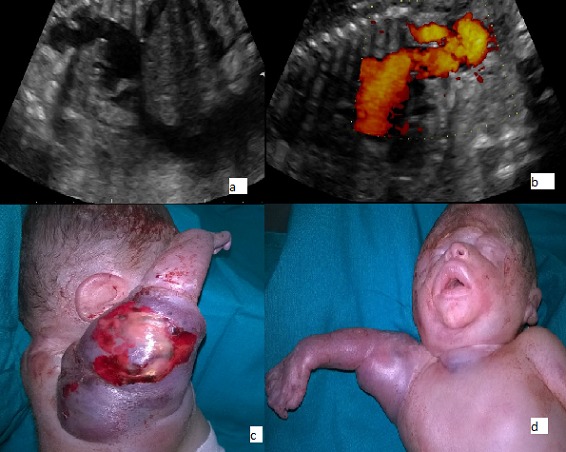
(A, B) doppler énergie montrait le départ de vaisseaux de l’aorte communiquant avec l’artère sous clavière droite; (C, D) tumeur vascularisée prenant et déformant tout le membre supérieur droit, allant jusqu’à la partie haute du thorax

